# Evaporation flow characteristics of airborne sputum droplets with solid fraction: Effects of humidity field evolutions

**DOI:** 10.1063/5.0076572

**Published:** 2021-12-02

**Authors:** Gang Zeng, Lin Chen, Haizhuan Yuan, Ayumi Yamamoto, Shigenao Maruyama

**Affiliations:** 1 School of Mathematics and Computational Science, Xiangtan University, Xiangtan 411105, People's Republic of China; 2 Institute of Engineering Thermophysics, Chinese Academy of Sciences, Beijing 100190, China; 3 University of Chinese Academy of Sciences, Beijing 100049, China; 4 National Institute of Technology, Hachinohe College, Hachinohe, Aomori 039-1192, Japan

## Abstract

The continuance of the COVID-19 pandemic largely depends on the spread of virus-carrying aerosols in ambient air. The mechanism of virus transmission and infection remains under intense investigation. In this study, an evaporation flow model of airborne sputum droplets is proposed which considers the evolution effects of the humidity field under different particle distributions and solid/salt fraction interactions. The incompressible Navier–Stokes equations characterize a stream of airflow jets, and the convection-diffusion-evaporation process is used to account for the inhomogeneous humidity field caused by the respiratory tract. Momentum equations for droplet dynamics which involve the effects of drag, gravity, and Brownian motion on sputum droplets are introduced to quantify the transport of droplets in a humidity field. The Lattice Boltzmann method is used to track the evolution of the aerosol in space and time under different ambient temperature and relative humidity conditions. The results of the simulation demonstrate that airborne humidity accelerates the evaporation rate of droplet, while supersaturated humid air forms a vapor mass in front of the respiratory tract. Despite the short lifespan of this phenomenon, it significantly hinders the evaporation of the droplets. Besides, the droplet vortex dynamics in a humidity field are sensitive to the droplet size.

## I. INTRODUCTION

The COVID-19 pandemic has attracted scientific attention to the mechanism of the transportation of airflow and the ejection of aerosols from the respiratory tract through coughing, sneezing, and breathing.^1–6^ Exhalation produces many turbulent clouds (including tiny droplets) with limited momentum, kinetic energy, and thermal energy, moving as a viscous fluid at an unstable drift velocity. These aerosols, which carry a small mass fraction of solid/protein/salt components, are transported or suspended in the air. Exploring the spread of these airborne droplets under different ambient conditions will help to understand the infection process and prevent the spread of the viruses.

In coughing, the accumulated air is compressed after deep breathing and then expelled rapidly. The exhaled air carries sticky saliva droplets from the lungs, respiratory tract, and trachea. Many studies have focused on the active forms of these clouds to determine the size of the sprayed droplets. For example, Bourouiba *et al.*^7^ investigated the fluid dynamics of expiratory flow and indicated that multiphase turbulent exhalation flow contains suspended droplets of various sizes. Bake *et al.*^8^ showed that the suspended particle diameter ranged from 0.01 to 1000.0 *μ*m, depending on the mechanism of their generation and their original site. Chao *et al.*^9^ measured the average diameter of the droplets using interferometric Mie imaging and found the average values of 13.5 and 16.0 *μ*m when coughing and talking, respectively.^9^ Yang *et al.*^10^ evaluated the size distribution of coughed droplets from people of different ages and genders by the aerodynamic particle sizer and the scanning mobility particle sizer system and reported that an average size distribution of the droplet nuclei was 0.58–5.42 *μ*m, where 82% of droplet nuclei were centered in a range of 0.74–2.12 *μ*m.^9^

Since the beginning of the COVID-19 pandemic, several studies have applied force balance analysis to aerosols in the airflow to explore the threat range of virus-carrying aerosols. Using molecular collision theory, a susceptible-exposed infectious recovered-deceased model for using in large-scale population exposure was proposed by Chaudhuri *et al.*^11^ They investigated the influence of the Stokes drag force on the dynamic transport of aerosols. The results indicate that the smaller droplet (<30.0 *μ*m) has a lower Stokes number, which allows it to float in the ambient air. However, that model ignored gravity, which may limit the effectiveness of its droplet trajectory predictions. Dbouk *et al.*^12^ investigated the transport, dispersion, and evaporation of saliva particles under the gravitational force and the Stokes drag force. A hexahedral non-uniform structured mesh was generated at the mouth-print, and the Weibull droplet distribution was used to simulate coughing. Regardless of wind speed, the water droplets fell to the ground less than 1.0 m away from the exhaling or coughing person, while saliva droplets traveled up to 6.0 m away under the effect of wind speeds from 4.0 to 15.0 km/h. However, ignoring the irregular Brownian motion of tiny suspended aerosols in the fluid does not obviously affect the characterization of droplet behavior. Das *et al.*^13^ used the Langevin equation to describe the diffusion and dissipation of tiny Brownian particles. They took into account the effects of drag, diffusion, and gravity on aerosols with different sizes and launch velocities. Their study indicated that the droplet with a radius of 100.0 *μ*m and initial velocities of 21.0, 10.0, and 5.0 m/s traveled 2.35, 1.1, and 0.55 m, respectively. Both the evaporation of the droplet and the exhaled airflow affect the movement of the droplet.

Computational fluid dynamics has been effectively used to study the flow of aerosols and in relation to different environments.^14,15^ Pendar *et al.*^16^ combined the compressible Navier–Stokes equations with a large eddy simulation turbulence model to simulate airflow. The numerical results indicated that the saliva droplets are transported to approximately 2.3 m at a velocity of 22.3 m/s in an average size of 90.0 *μ*m; when the droplet size reaches 540.0 *μ*m, the droplet may be transported to more than 4.0 m. However, this model ignored the temperature effect. Li *et al.*^15^ applied a k-ε turbulent model to simulate a cough and added the mass and turbulent kinetic energy consumed by droplet evaporation as source terms for the continuity, momentum, and energy equation, and found that the projection distance between the millimeter-level droplet in the still air may reach more than 1.0 m. This model increased the calculation load and it ignored the humidity field, which could slow the evaporation of droplets. Rosti *et al.*^2,3^ solved the incompressible Navier–Stokes equations using direct numerical simulation to model airflow, and the advection-diffusion equation was also employed to describe the humidity field. Nevertheless, the influence of temperature on relative humidity (RH) was not considered in this model. Hydrodynamic methods have also been introduced recently to explore the mechanism of pathogens and provide new information to prevent viral spread.

Several models^11–15^ have been developed which focus on the effects of respiratory jets, air-airflow interaction, droplet dynamics, and environmental humidity. However, most of those models ignore airborne infection which originates from the inhomogeneous humidity field caused by the saturated water vapor exhaled from the respiratory tract. The exhaled airflow can be assumed to be saturated or close to the saturation line.^2,17^ The droplets ejected from the respiratory tract (particularly for aerosols below 5.0 *μ*m) are wrapped in a vapor cloud of saturated humidity and showed various dynamic behaviors. For example, Mahjoub *et al.*^18^ visualized the shape and propagation of the exhaled airflow and its interaction with the ambient air by using high-speed photography, schlieren photography, and PIV (Particle Image Velocimetry). However, the interaction between the omitted temperature and the humidity field has been ignored. Li *et al.*^19^ proposed a multi-component Eulerian model in which the moist air is treated as an ideal mixture of the dry air and vapor, and separately solved the continuity equation of the components. Actually, this is not a multi-component model composed of a two-phase mixture of gas and liquid, and the droplet is dispersed into a fluid phase which is hotter and moister than that of the ambient air.^4^ In addition, the transmission characteristics of airborne water vapor cannot be ignored, which are affected by many factors, such as temperature and the vapor diffusion coefficient.

This study proposes an evaporation flow model of airborne sputum droplets containing solid/protein/salt ingredients and presents an efficient and accurate numerical method to simulate the dynamic behaviors of the dispersion and evaporation flow of airborne sputum droplets in a humidity field. This model features an advection-diffusion equation that describes the evolution of the supersaturation field puffed from the respiratory tract. The classic Eulerian–Lagrangian method based on one-way coupled fluid dynamics was used to combine the complex flow processes, including the ejection of an incompressible fluid containing droplets, energy balance, mass transfer, and dynamic transport. Physical parameters, such as ambient temperature and RH, are simulated by the Lattice Boltzmann method. The vortex dynamics of droplets in the humidity field are investigated by integrating the Langevin equation with gravity and drag force. The group of droplets obeying the Rosin–Rammler distribution in humidity field are discussed in detail.

## II. METHODOLOGY

### A. Physical droplet flow

**FIG. 1.**
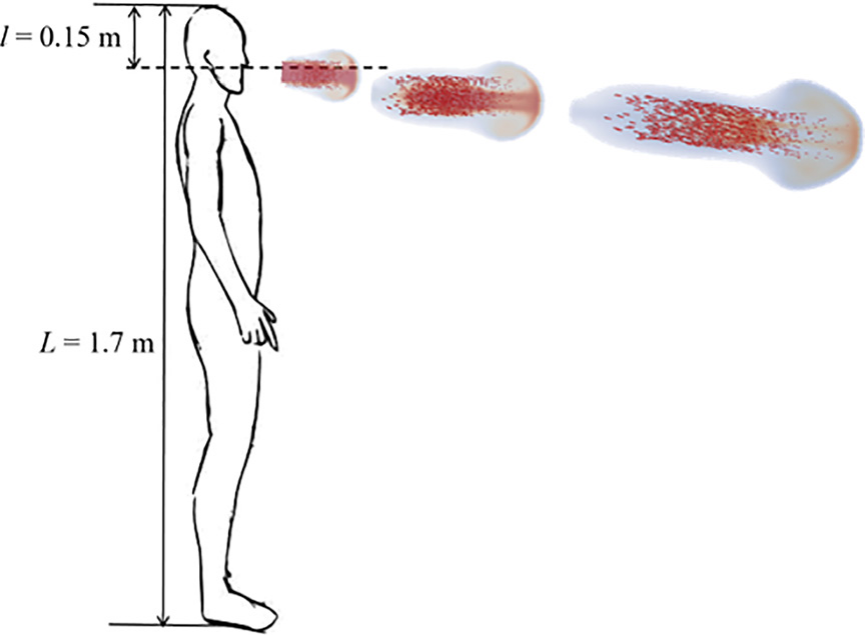
The geometry of the air mass released by coughing from the lips. The height of the cougher is 1.7 m.

In this study, the streams of humid jets emitted in talking, coughing, and sneezing are simulated. An aerosol with radius *r* is ejected with initial horizontal velocity *u_0_*. As shown in Fig. 1, a person with a height of 1.7 m exhales a saturated droplet cloud with an initial temperature of 36.0 °C, and the humidity of the mouth area is close to saturation (RH = 1.0). The temperature of the airflow and the droplet are *T_f_* and *T_d_*, respectively. In evaporative flow from sneezing or coughing, aerosols carrying solid/protein/salt components finally settle on the ground or suspend in the air. The gravitational force, the Stokes drag force, and the Brownian force have an effect on the dynamic behavior of droplets.

### B. Detail model formulations

#### 1. Cough*-*generated airflow model

In order to develop a mathematical model of the droplet dynamics in a turbulent airflow exhaled from the mouth, the incompressible Navier–Stokes equations system is used to model the ambient airflow field.^1–5^ Considered with the tiny suspended aerosols (diameters smaller than the Kolmogorov scale^20^) the collisions between droplets and the force feedback on the airflow can be ignored.^2,3,21^ The continuity and momentum equations used in this model are defined as follows:

$$\{\begin{array}{c} \nabla \cdot u_{f}=0\begin{array}{c} , \end{array}\\ \rho_{f}(\frac{\partial u_{f}}{\partial t}+u_{f}\cdot \nabla u_{f})=-\nabla p+\mu_{f}\nabla^{2}u_{f}, \end{array}$$ (1)

where **u_*f*_** (m/s), *ρ_f_* (kg/m^3^), and *p* (Pa) represent the velocity, density and pressure field, respectively, and *μ_f_* (Pa·s) is dynamic viscosity coefficient of airflow. The droplets produced in the respiratory tract are carried out by the respiratory airflow, and the evolution of the aerosol radius is significantly affected by the temperature of the airflow surrounding the droplet. The initial temperature of the airflow is close to the body temperature of the human body. The movement of the exhaled airflow into the air is accompanied by the transfer of energy. The energy equation used to describe the temperature change of the airflow can be given as

$$\frac{\partial T_{f}}{\partial t}+u_{f}\cdot \nabla T_{f}=\frac{\lambda }{\rho_{f}c_{p}}\Delta T_{f}.$$ (2)

Here, *T_f_* (°C) is the temperature field of airflow, *c_p_* [J/(kg·°C)] and *λ* represent the specific heat capacity and the thermal conductivity coefficient, respectively.

Except the temperature of the airflow, the humidity around the droplet determines the amount of saturated water vapor on the surface of the droplet and affects the evaporation of the droplet. The exhaled airflow is assumed as saturated, or close to saturation.^2,17^ The moving airflow mixes with the air, and gradually becomes unsaturated. The advection-diffusion equation is used to describe the evolution of supersaturation field,^1,3,22^ which is written as

$$\frac{\partial \varphi }{\partial t}+u_{f}\cdot \nabla \varphi =D_{d}\Delta \varphi ,$$ (3)

where *φ* (= RH − 1) is supersaturation field, and *D_d_* is the water vapor diffusion coefficient. The fluctuations of supersaturation are produced inside the cloud by the turbulent velocity field acting on the vertical gradient of humidity sustained by temperature.^22^ The droplet clip is wrapped in a cloud. The humidity of the cloud determines the humidity on the surface of the droplet and its evaporative flow.

#### 2. Droplet evaporation model

##### a. Basic evaporation model

The evaporation of droplets is governed by the amount of evaporation of the droplet vapor into the air (*Va*), which is mainly determined by the difference between the amount of saturated steam on the droplet interface (*C*_1_ [kg/m^3^]) and the amount of partial steam in the air (*C*_2_ [kg/m^3^]). The expression is given as

$$Va=k(C_{1}-C_{2}),$$ (4)

where *Va* [kg/(m^2^·s)] is the amount of the evaporation per unit time and surface area and *k* (m/s) is the mass transfer coefficient. Water activity is a thermodynamic measure of water, which expressed as the vapor pressure of water in a sample divided by vapor pressure of pure water at a given temperature.

##### b. Solid fraction of the saliva droplet

For pure water droplets, it can be assumed that the surrounding vapor of the droplet interface is saturated. However, the droplet exhaled from talking, coughing, and sneezing is not pure water, which contains dissolved substances, such as salt and protein. In this case, the solid/salt and other parts should be considered in the droplet evaporation process. In this study, the experimental measurements of saliva samples were collected from 9 students (4 Male, 5 Female) living in Aomori Prefecture of Japan, with an average age of 20. For each students, 1.0 ml of saliva was collected into vials using micropipette. Then the saliva samples were dried at 60 °C for 90 h. The weight for different statuses was conducted by electronic scale (GR-202; A&D, Ltd.,). The results are shown in Table I. It can be seen that the average weight of the left solid component in the saliva is 0.7621 ± 0.0016 wt. %. According to that, the solid fraction in the saliva droplet is defined as the same value in the present numerical model. Employing the evaporation flow model with the evaporation of the droplet in the flow field, the droplet has an equilibrium state that the system stops evaporation and becomes a floating droplet in the flow, though it has a dynamic equilibrium state with fluctuations of temperature and evaporation-condensation process in the interface of the droplet.

**TABLE I.** Experimental data of saliva samples.

Sample	1	2	3	4	5	6	7	8	9	Ave	Med	SD
Age	18	19	19	19	20	20	21	22	22	20	20	
Male/female	F	F	F	M	M	M	F	M	F	⋯		
Vial (g)	23.4681	23.4681	23.9263	23.6836	23.7829	23.4606	23.428	23.4313	23.5069	⋯		
Vial + Saliva (g)	24.4613	24.4684	24.9315	24.6845	24.786	24.4475	24.4259	24.4259	24.5047	⋯		
Saliva (g/ml)	0.9932	1.0003	1.0052	1.0009	1.0031	0.9869	0.9979	0.9946	0.9978	0.9978	0.9979	0.0053
Vial + Dry weight of saliva	23.4790	23.4741	23.9327	23.6928	23.7901	23.4688	23.4334	23.4394	23.5139	⋯		
Dry weight of saliva (g)	0.0109	0.0060	0.0064	0.0092	0.0072	0.0082	0.0054	0.0081	0.0070	0.0076	0.0072	0.0016
wt% of solid component	1.097	0.600	0.637	0.919	0.718	0.831	0.541	0.814	0.702	0.7621	0.7178	0.1637
wt% of liquid component	98.903	99.400	99.363	99.081	99.282	99.169	99.459	99.186	99.298	99.2379	99.2822	0.1637

Under the above conditions, the saturation pressure of water is reduced by the nonvolatile components, which affects the evaporation rate of the droplets. Based on Raoult's law,^23,24^ this study describes the amount of water vapor *C*_1_ on the saliva surface as the following:

$$C_{1}=x_{v}\frac{0.217P_{\mathrm{sat},T_{d}}}{\left(T_{d}+273.15\right)},$$ (5)

where *T_d_* (°C) is droplet surface temperature, *x_v_* refers to the mole fraction of evaporated solvent in saliva, and $P_{\mathrm{sat},T_{d}}$(Pa) is the saturated water vapor pressure on the interface of the droplet (Appendix). *C*_2_ is closely related to the partial water vapor pressure in the air,

$$C_{2}=\frac{0.217P_{\mathrm{sat},T_{f}}\text{RH}}{\left(T_{f}+273.15\right)},$$ (6)

where *T_f_* (°C), RH, and $P_{\mathrm{sat,}T_{f}}$(Pa) are ambient air temperature, relative humidity, and water vapor pressure of airborne (Appendix), respectively. The mass transfer coefficient *k* is correlated with the Schmidt number (Sc), Reynold number (Re), and the diffusion coefficient (*D* [m^2^/s]) of vapor in the air,

k=DDd(2.0+0.6Re1/2Sc1/3), (7)

where *D_d_* (m) is the droplet diameter. The mass transfer equation of the droplet can be derived as

$$\frac{dm_{d}}{dt}=-A_{d}Va,$$ (8)

where *m_d_* (kg) is the droplet mass, and *A_d_* (m^2^) is the surface area of the droplet. The mass transfer of droplet is mainly reflected in the change of droplet radius. Therefore, the mass of the droplet is divided as

$$\frac{dr}{dt}=-\frac{Va}{\rho_{d}}.$$ (9)

The droplet temperature is governed by the thermal balance. By ignoring the radiation heat exchange, the heat exchange between the droplets and air mainly includes the convective heat exchange and the evaporative heat exchange. Therefore, the heat transfer equation can be written as^11,15^

$$m_{d}C_{pd}\frac{dT_{d}}{dt}=hA_{d}(T_{f}-T_{d})+L\frac{dm_{d}}{dt},$$ (10)

where *L* (J/kg) is the latent heat of the droplet. The convective heat transfer coefficient *h* [W/(m^2^·K)] is calculated by a modified Nusselt number as

$$h=\frac{\lambda In(1+B_{T})}{D_{d}B_{T}}(2+0.6{\text{Re}}_{d}^{0.5}{\mathrm{Pr}}^{1/3}),$$ (11)

where Pr is the Prandtl number and *λ* is the thermal conductivity coefficient of air. The Spalding heat transfer number (*B_T_*) can be calculated by^15^

$$B_{T}=\frac{C_{pv}(T_{f}-T_{d})}{L-\frac{q_{d}}{{\dot{m}}_{d}}},$$ (12)

where ${\dot{m}}_{d}$ is the droplet evaporation rate, *q_d_* (J) is the heat energy transferred to the droplet amd *C_pv_* [J/(kg·°C)] is specific heat.

#### 3. Droplet tracking model

The small aerosols (the particle diameter is generally 10^−7^–10^−5^ m) suspended in the airflow keep a random irregular Brownian motion, which is the interaction between Brownian particles and the surrounding medium. The Langevin equations are written as^13,25–27^

$$\frac{dx_{d}}{dt}=u_{d},$$ (13)

$$m_{d}\frac{du_{d}}{dt}=-\lambda u_{d}+\zeta (t),$$ (14)

where d**x**_*d*_/d*t* and d**u**_*d*_/d*t* are the coordinate and velocity change in each discrete time step, respectively, and **x**_*d*_ (m) and **u**_*d*_ (m/s) represent the Cartesian components of the position and velocity vectors. The first term on the right-hand side of Eq. (14) is the frictional force, which is proportional to the velocity of the Brownian particle.^25^ Among them, the friction coefficient is given by Stokes law, *λ* = 6π*μ_f_R_d_*, where *R_d_* (m) is the droplet radius and *μ_f_* (Pa·s) is the viscosity.

The second term in the right-hand side of Eq. (14) represents the fluctuating force which is supposed to the accidental collision between the Brownian particle with molecules of the surrounding medium.^28^ The effect of the fluctuating force can be summarized by giving a Gaussian random function with the first-order moment of 0 (⟨*ζ*(*t*)⟩ = 0) and a second-order moment of *D* (⟨*ζ*(*t*), *ζ*(*t′*)⟩= *Dδ*(*t* − *t′*)).^28^ In the expression, *D* is the strength of the fluctuating force, which is obtained from the Einstein relation, *D* = *K_B_T_d_λ*, where *K_B_* = 1.38 × 10^−23^ J/K is the Boltzmann constant.

The Langevin equations mentioned above only contain the force which expresses the Brownian motion of the tiny droplet. To involve the effect of other forces, the Langevin equations can be further written as

$$\begin{array}{c} m_{d}\frac{du_{d}}{dt}=(\rho_{d}-\rho_{f})gV_{d}-\frac{3}{4}C_{d}\frac{\rho_{f}}{\rho_{d}}\frac{m_{d}}{2R_{d}}|u_{d}-u_{f}|(u_{d}-u_{f})\\ +\frac{\rho_{f}V_{d}}{2}\frac{\partial (u_{f}-u_{d})}{\partial t}-\lambda u_{d}+\zeta (t). \end{array}$$ (15)

The first three terms on the right-hand side of Eq. (15) represent the gravitational force, the Stokes drag force, and the buoyancy force, respectively. *m_d_* (kg), *V_d_* (m^3^)*, R_d_* (m)*, ρ_d_* (kg/m^3^), and ***u***_*d*_ (m/s) are the mass, volume, radius, density, and velocity of the saliva droplets, respectively. *ρ_f_* (kg/m^3^) and ***u***_*f*_ (m/s) are the density and velocity of airflow, respectively. *C_D_* is the drag coefficient which can be calculated by a function of Reynold number as

$$C_{D}=\{\begin{array}{l} \frac{24}{\text{Re}}\begin{array}{cc} & (0<\text{Re}<1.2^{\frac{10}{3}}), \end{array}\\ \frac{20}{{\text{Re}}^{0.7}}\begin{array}{cc} & (1.2^{\frac{10}{3}}<\text{Re}<32), \end{array}\\ \frac{10}{\sqrt{\text{Re}}}\begin{array}{cc} & (32<\text{Re}<500), \end{array} \end{array}$$ (16)

$$\text{Re}=\frac{\rho \left|u_{d}-u_{f}\right|D_{d}}{\mu }.$$ (17)

### C. Numerical algorithm

The present work refers to fluid flow, heat transfer, droplet movement and droplet evaporation. To further improve the computational accuracy, an effective method based on OpenMP technology has been employed in this section. A comparison between the current model and literature models can be found in Table II.

**TABLE II.** Summary of basic model considerations of the current and previous studies.

Reference	Particle distribution	Solid fraction	Evaporation flow interaction	Evaporation model	Energy balance	Drag force	Gravity	Brownian motion
Current model	√	√	√	√	√	√	√	√
Rosti *et al.*^3^	⋯	⋯	√	√	⋯	√	√	√
Chaudhuri *et al.*^10^	⋯	√	⋯	√	√	√	⋯	⋯
Dbouk *et al.*^11^	√	⋯	⋯	√	√	√	√	⋯
Das *et al.*^12^	⋯	⋯	⋯	⋯	⋯	√	√	√
Pendar *et al.*^14^	√	⋯	⋯	⋯	⋯	√	√	⋯
Li *et al.*^15^	⋯	√	√	√	√	√	√	⋯
Kumar *et al.*^22^	√	⋯	⋯	√	√	√	√	√

#### 1. Lattice Boltzmann method for flow field

Unlike the traditional CFD numerical methods, the lattice Boltzmann method (LBM) has the characteristic of the mesoscopic model between the microscopic molecular dynamics model and the macroscopic continuous model. LBM is used to solve Eq. (1) due to its advantages of simple boundary setting, easy parallelization, and simple program implementation. Equations (19) and (20) approximately recover the Navier–Stokes equations to the second order accuracy through the Chapman–Enskog expansion. The LB equation can be written as^29,30^

$$f_{\alpha }^{*}(x,t)=f_{\alpha }(x,t)-\frac{1}{\tau }(f_{\alpha }(x,t)-f_{\alpha }^{eq}(x,t)),$$ (18)

$$f_{\alpha }(x+e_{\alpha }\Delta t,t+\Delta t)=f_{\alpha }^{*}(x,t),$$ (19)

with

$$f_{\alpha }^{eq}=\omega_{\alpha }\rho_{f}[1+\frac{e_{\alpha }\cdot u_{f}}{c_{s}^{2}}+\frac{{\left(e_{\alpha }\cdot u_{f}\right)}^{2}}{2c_{s}^{4}}-\frac{u_{f}^{2}}{2c_{s}^{2}}].$$ (20)

Here, *f_α_* and *f^eq^_α_* are the distribution function and the equilibrium distribution function, respectively. *τ* is the single relaxation parameter, *Δt* is the time interval, ***e***_*α*_ and *ω_α_* represent the particle velocity and the weights coefficient, respectively. For D3Q15 model, *ω_α_* and *c*^2^_*s*_ are set as *ω*_0_ = 16/72, *ω*_1–6_ = 8/72, *ω*_7–14_ = 1/72, and *c*^2^_*s*_ = *c*^2^/3, respectively. The particle velocity of D3Q15 is given as

$$e_{\alpha }=c\{\begin{array}{c} (0,0,0)\\ \left(\pm 1,0,0),(0,\pm 1,0),(0,0,\pm 1\right)\\ (\pm 1,\pm 1,\pm 1) \end{array}\begin{array}{c} \alpha =0\begin{array}{c} , \end{array}\\ \alpha =1-6\begin{array}{c} , \end{array}\\ \alpha =7-14, \end{array}$$ (21)

where *c* = *Δx/Δt *=* *1 is the lattice velocity and *Δx* is the lattice spacing. The kinematic viscosity *υ*, which is related to the relaxation time *τ*,

$$v=c_{s}^{2}(\tau -0.5)\Delta t.$$ (22)

The fluid density *ρ* and velocity ***u***_*f*_ are computed by the density distribution function *f_α_* as

$$\rho_{f}=\sum_{\alpha }f_{\alpha },$$ (23)

$$u_{f}=\frac{1}{\rho_{f}}\sum_{\alpha }e_{\alpha }f_{\alpha }.$$ (24)

#### 2. Computational sequence and model setting

In this study, a person with a height of 1.7 m is considered to stand in an outdoor environment, and breathe, cough, or sneeze droplets that is approximately 36.0 °C. The oral cavity is 1.55 m above the ground (Fig. 1). The exhaled turbulent cloud only affects the small area outside the lips, and then dissipates and merges into the air with a short time.^12,31^ Shrinking the turbulent cloud cluster area (the physical domain is 3.2 × 0.6 × 0.6 m^3^, and the grid is set to 800 × 150 × 150) improves the calculation efficiency. The center position of the lips is (0, 0.3, and 0.3). A ghost cell approach is used to impose the boundary conditions. The inlet velocity is set to the Dirichlet boundary condition, and the velocity is only imposed on the lips. After completing an exhalation, the inlet boundary velocity is changed to zero. The Neumann outflow boundary conditions are used for the rest boundaries.

The droplet is initially at rest and randomly distributed over a 4.0 × 4.0 cm mouth plane. The droplet and the turbulent cloud are injected at the same velocity from the mouth into the computational domain with an airborne temperature *T_f_*  = 30.0 °C and RH = 0.5 (i.e., *φ* = −0.5). The velocity of the mouth in 0.16 s^12^ is set to *u_x_* = 5.5 m/s. The kinematic viscosity coefficient of the air is set to the order of 10^−6^ m^2^/s. The resulting Reynolds number (based on the exhalation velocity and the mouth average diameter) is about 6000. The initial droplet size distribution is based on the Rosin–Rammler distribution law,^32,33^ which is widely used to represent the droplet size distribution in sprays.^34^ The total number of emitted cough droplets is set to 1500, and each droplet is composed of 99.2379% water and 0.7621% salt.

**FIG. 2.**
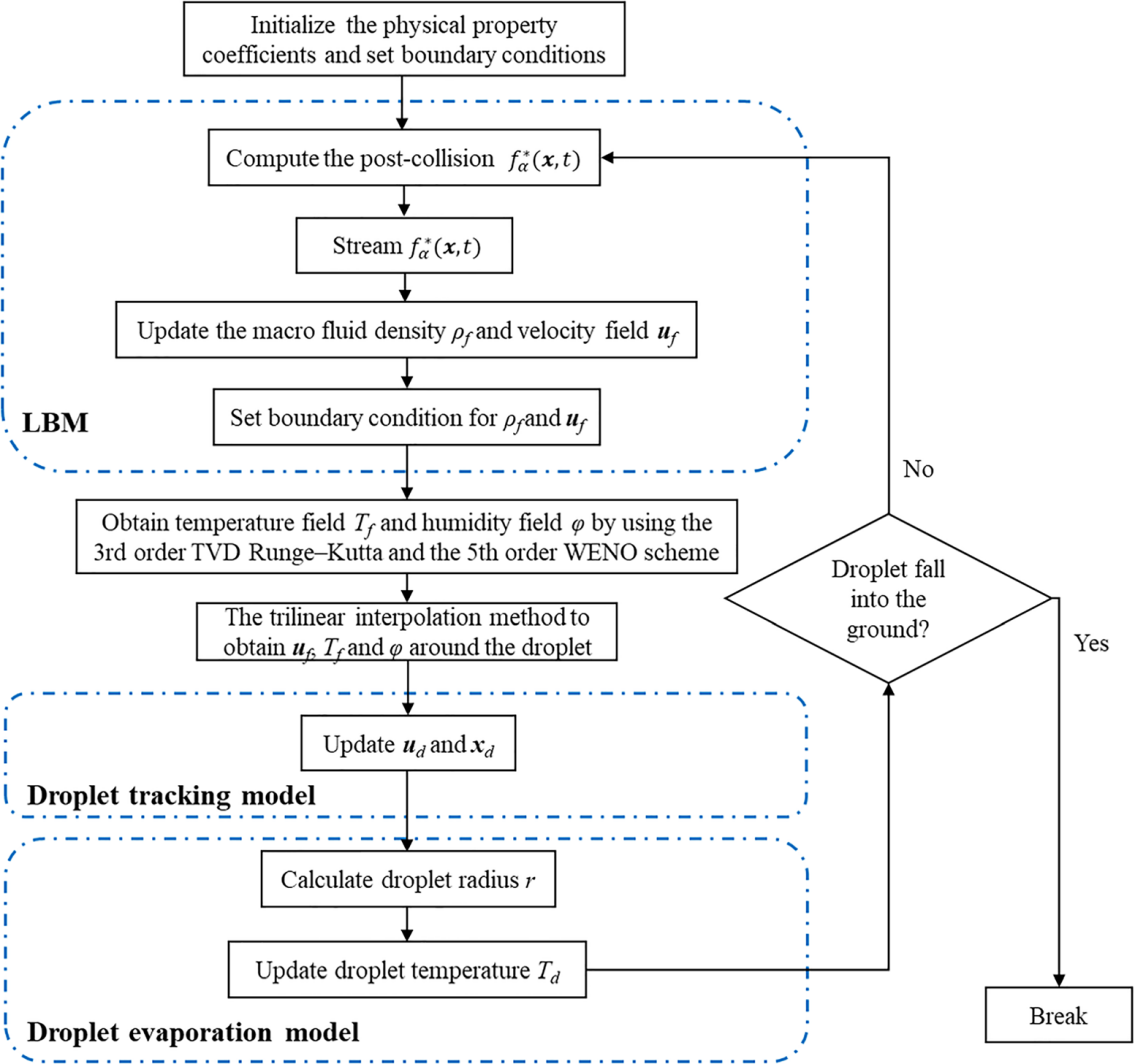
Computational sequence for the droplet evaporation simulation.

The algorithm is based on the Lattice Boltzmann method for the discretization of the Navier–Stokes equations [Eqs. (1) and (18)–(24)] on Euler grids. The third-order explicit TVD Runge–Kutta method and the fifth-order high-precision WENO scheme are used to discretize the time and space, respectively, which can accurately calculate the exhaled temperature field and humidity field [Eqs. (2) and (3)]. Droplet dynamics are described in the Lagrangian coordinates, and the governing equations for the droplet evaporation [Eqs. (8) and (10)] and the droplet tracking [Eqs. (13)–(15)] are evolved by using the low-storage third-order TVD Runge–Kutta scheme. The trilinear method of interpolation is used to obtain the humidity, velocity, and temperature surrounding the aerosol, which achieves one-way coupling^34^ between turbulent clouds and droplets. The humidity field is used as the ambient humidity together with the amount of saturated water vapor on the interface of the droplet achieving mass couple by calculating the amount of partial steam in the airborne. Momentum coupling is the result of the Stokes drag force integrated the dispersed with continuous phase. Energy coupling occurs through heat transfer between phases. The temperature obtained by interpolation is used as droplet interface temperature to calculate the convective heat exchange between the air and the droplet. The detailed computational sequence is shown in Fig. 2.

## III. RESULTS AND DISCUSSION

### A. Validation of the droplet evaporation model

**FIG. 3.**
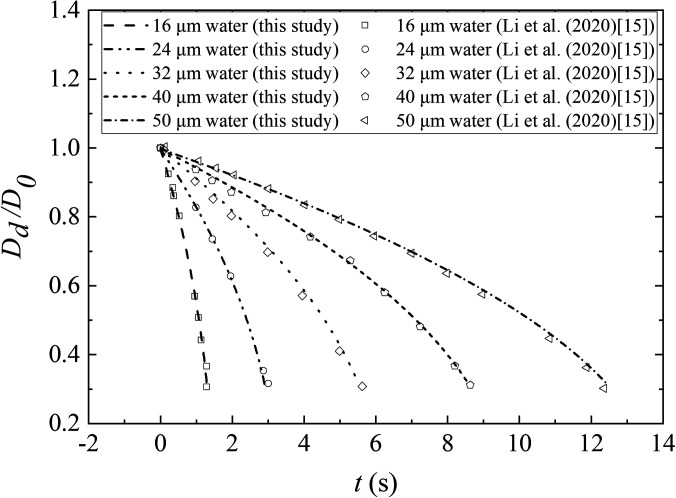
Evaporation of small droplet of pure water at different times, comparing with the results obtained by Li *et al.*^15^ The ambient air temperature is 30.0 °C with RH = 0.84. The droplet initial temperature is 36.0 °C.

In this section, the existing single pure droplet evaporation is compared with the numerical results of Li *et al.*^15^ to validate the effectiveness of the present model. The pure water droplet is set in five different diameters (16.0, 24.0, 32.0, 40.0, and 50.0 *μ*m) to compare their evaporation time in a moderate temperature environment. The ambient air temperature and the initial droplet temperature are 30.0 °C and 36.0 °C, respectively, and RH is 0.84. In Fig. 3, A good agreement between the present results with the published data.^15^

### B. Effect of relative humidity and humidity field evolutions

**FIG. 4.**
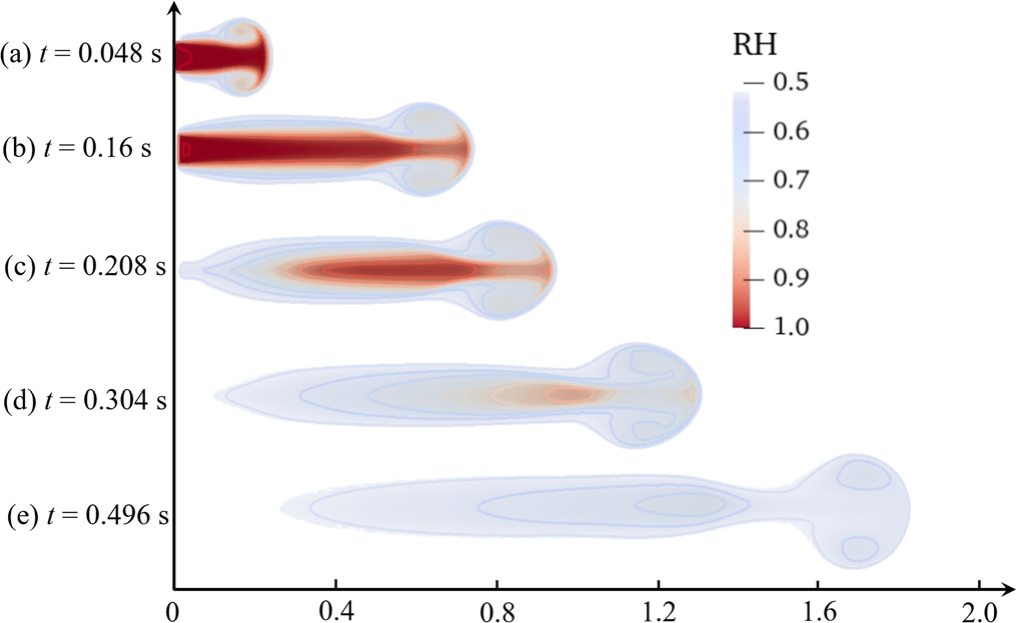
The contour of humidity field with vapor concentration. The ambient air temperature and the initial droplet temperature is 36.0 °C and 30.0 °C, respectively. The air humidity is 0.5. (a) *t *=* *0.048; (b) *t *=* *0.16; (c) *t *=* *0.208; (d) *t *=* *0.304; and (e) *t *=* *0.496 s.

The above validation is conducted on pure water in a coordinated environment (stable RH), which ignores the effect of the inhomogeneous humidity field.^35^ However, a cough is characterized by the instantaneous pulsed turbulent cloud carrying water vapor and droplets from the respiratory tract, which significantly increases the local airborne humidity, and the vapor concentration is significantly beyond its level in airborne. The humidity field with vapor concentration contour is shown in Fig. 4. The ambient air temperature and the initial droplet temperature are 36.0 °C and 30.0 °C, respectively, and the air humidity is 0.5. Turbulent clouds from the humidity field diffuse with the unsaturated airborne along the direction of velocity, which quickly dissipated at *t *=* *0.496 s, only a slight vapor concentration gradient can be observed. Due to the exhalation of supersaturated air and the evaporation of droplets, respiratory droplets are dispersed in an inhomogeneous humidity field, particularly in the area near the opening respiratory tract (such as the lips and nostrils). Therefore, the RH and the humidity field have significant effects on the characteristics of droplet evaporation, droplet dispersion trajectories, and viral viability.

**FIG. 5.**
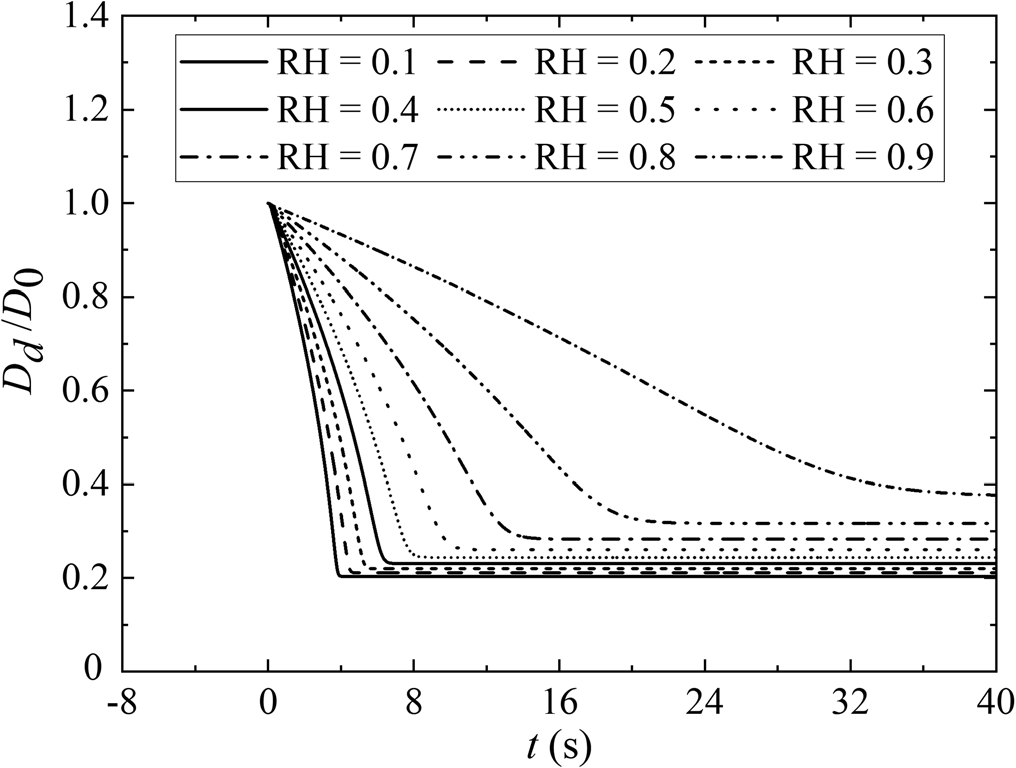
The evaporation rate for airborne sputum droplets with solid fraction at different realtive humidity. The ambient air temperature and the initial droplet temperature is 36.0 °C and 30.0 °C, respectively. The expiratory velocity is 5.5 m/s with *D*_0_ = 80.0 *μ*m.

RH refers to the percentage of water vapor pressure in the ambient air to the saturated water vapor pressure at the same temperature. The evaporation rate for airborne sputum droplets with a solid fraction at different RH (from 0.1 to 0.9) is compared in Fig. 5. The ambient air temperature and the initial droplet temperature is 36.0 °C and 30.0 °C, respectively. The expiratory velocity is 5.5 m/s and the initial droplet diameter is 80.0 *μ*m. The evaporation time before the droplet transforms into an aerosol increases with RH. By the inspection, the evaporation time for a droplet at RH = 0.9 is approximately 35.0 s, which is five times longer than that at RH = 0.5. The greater the air humidity, the greater amount of airborne saturated water vapor, which makes the droplets evaporate quickly. Under a lower RH, a small droplet evaporates rapidly into smaller residual nuclei, which remains suspending for a long time. Therefore, pathogens within the nuclei of these droplets may present a greater long-range transmission threat than the droplets in high humidity.

RH not only influences the rate of droplet evaporation but also determines the equilibrium state of droplet evaporation. When the droplet evaporates to a certain extent, an inflection point appears, and droplet diameter maintains an equilibrium stage (Fig. 5). Similar numerical result has been reported in the literature.^15,37^ In the case with humidity of 0.9, the droplet diameter is reduced to 29.8 *μ*m at most, which is almost twice the time's value of that at a humidity of 0.1 (16.3 *μ*m). The reason is that these droplets contain a variety of complex and nonvolatile soluble components, such as salt and protein, which decrease the activity of water. The mole fraction of the solvent in saliva decreases with the decreasing in moisture. Conversely, the proportion of droplet vapor evaporated into the air decreases with the increasing mole fraction of the solute. The presence of solid components in the aerosol increases the latency of the virus immersed in droplets.

**FIG. 6.**
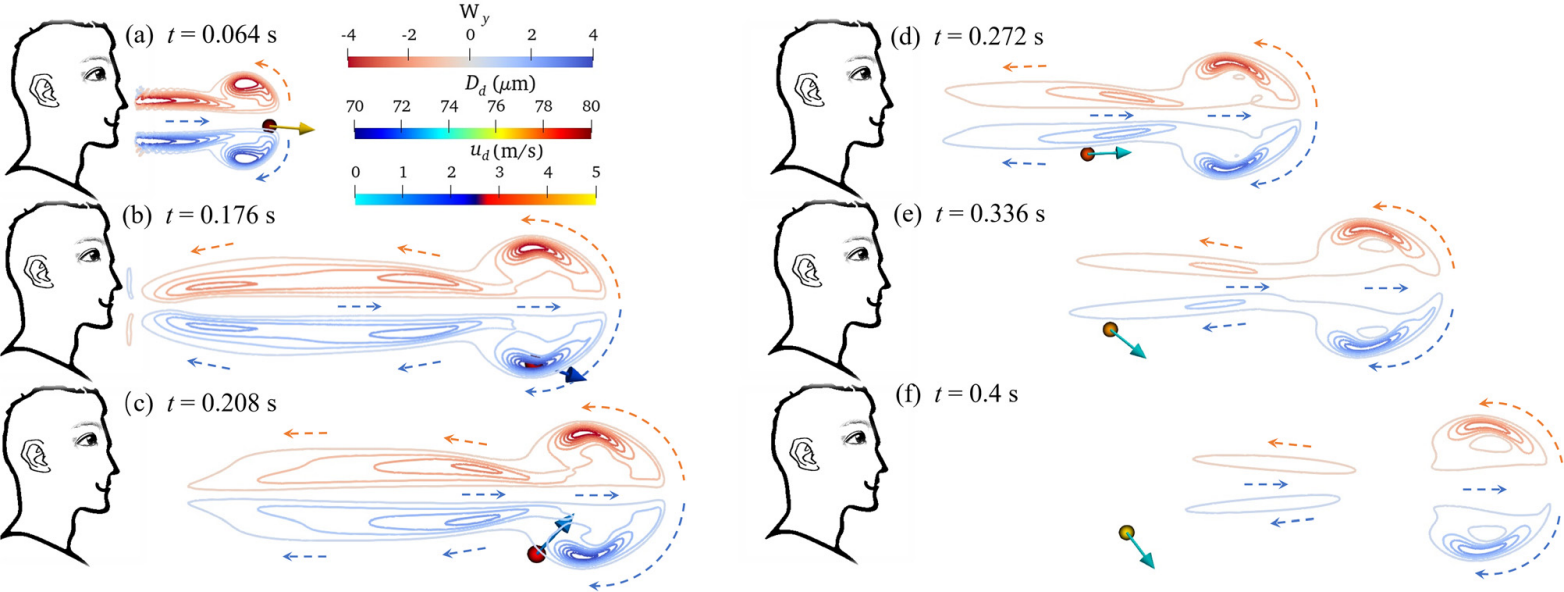
The vorticity field on the x-z plane and the motion of a single drop in a humidity field: (a) *t *=* *0.064, (b) *t *=* *0.176, (c) *t *=* *0.208, (d) *t *=* *0.272, (e) *t *=* *0.336, and (f) *t *=* *0.4 s. [The vorticity calculation expression is: $W=(W_{x},W_{y},W_{z})=\nabla \times u_{f}$.]

Violent expiratory events, such as coughing and sneezing, generate turbulent jets.^36^ The vortex produced in a breathing cycle can be observed in Fig. 6. The vorticity calculation as $W=(W_{x},W_{y},W_{z})=\nabla \times u_{f}$. Figure 6(a) indicates that the initial growth of turbulent jets is a direct consequence of large-scale motions generated at the jet boundaries, and these large-scale motions are primarily responsible for jet production and the initial entrainment of ambient fluid.^35^ The rapid jetting turbulent cloud cluster and the outside stationary air produce an unstable shear flow due to their difference in velocity field; further, a ring vortex is formed at the front, and a jet is formed at the tail.^31^ The numerical results of the vorticity are consistent with the results in the literature.^31^ During the exhalation (0–0.16 s), the ring vortex constantly increases under the effect of the turbulent jet flow force, and the ring vortex enters the irrotational ambient fluid and then rolls the irrotational ambient fluid into the jet [Fig. 6(b)]. After one exhalation is completed, the tailing jet fails to provide enough kinetic energy for the leading vortex, which is extremely unstable and begins to falloff [Fig. 6(f)]. The tailing jet will also form a small vortex. As the environmental ambient fluid is continuously involved in the ring vortex, which could not provide enough kinetic energy to support the trailing jet, and thus its velocity gradually decreases and disappears, while the vorticity disappears.

**FIG. 7.**
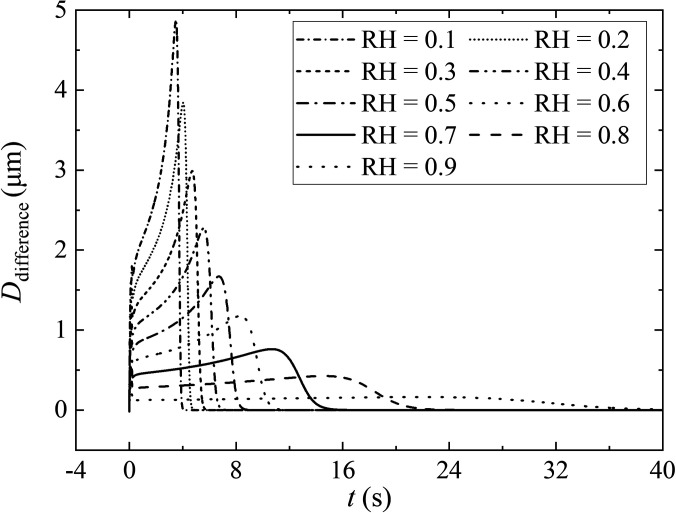
Comparison of the influence of humidity field on the evolution of droplet size. *D*_different_ is the difference between the droplet diameter under the humidity field and the non-humidity field at the same time. The ambient air temperature and the initial droplet temperature are 36.0 °C and 30.0 °C, respectively. The expiratory velocity is 5.5 m/s with *D*_0_ = 80.0 *μ*m.

The humidity field, which originates from the turbulent velocity field, and acts on the vertical gradient of humidity, is sustained by temperature. The existence of the humidity field depends on the velocity field. It can be observed in Figs. 4(e) and 6(f) that the disappearance of the vorticity happens with the disappearance of the humidity field. Within a few seconds of coughing, the droplets ejected from the respiratory tract are all located in the moist turbulent cloud, which can hinder their evaporation. The distribution color of the droplet diameter [Figs. 6(a)–6(d)] can be observed this phenomenon. Qualitative results of the humidity field for droplet evaporation are shown in Fig. 7. *D*_different_ is the difference between the droplet diameter under the humidity field and the non-humidity field at the same time. Generally, the higher the RH is the smaller influence on the humidity field applied on droplet evaporation. At the beginning of the exhalation (within 0.16 s), *D*_different_ is 1.6 *μ*m at RH = 0.1. At this time, the diameter of the droplet under the non-humidity field only evaporates within 2.0 *μ*m. It indicates that the droplet is greatly protected compared to the droplet in a non-humidity field. From the peak value of *D*_different_ at different levels of humidity, the lower the environmental humidity could result the higher the degree of protection of the droplets. After an exhalation is complete, the influence of the humidity field on the evaporation of droplets gradually decreases, but it still continues. *D*_different_ gradually expands. The maximum value of *D*_different_ nearly reaches 5.0 *μ*m for RH = 0.1. With the gradual dissipation of the humidity field, the influence of humidity field on droplet evaporation is disappeared, and the droplet is in the same equilibrium state.

**FIG. 8.**
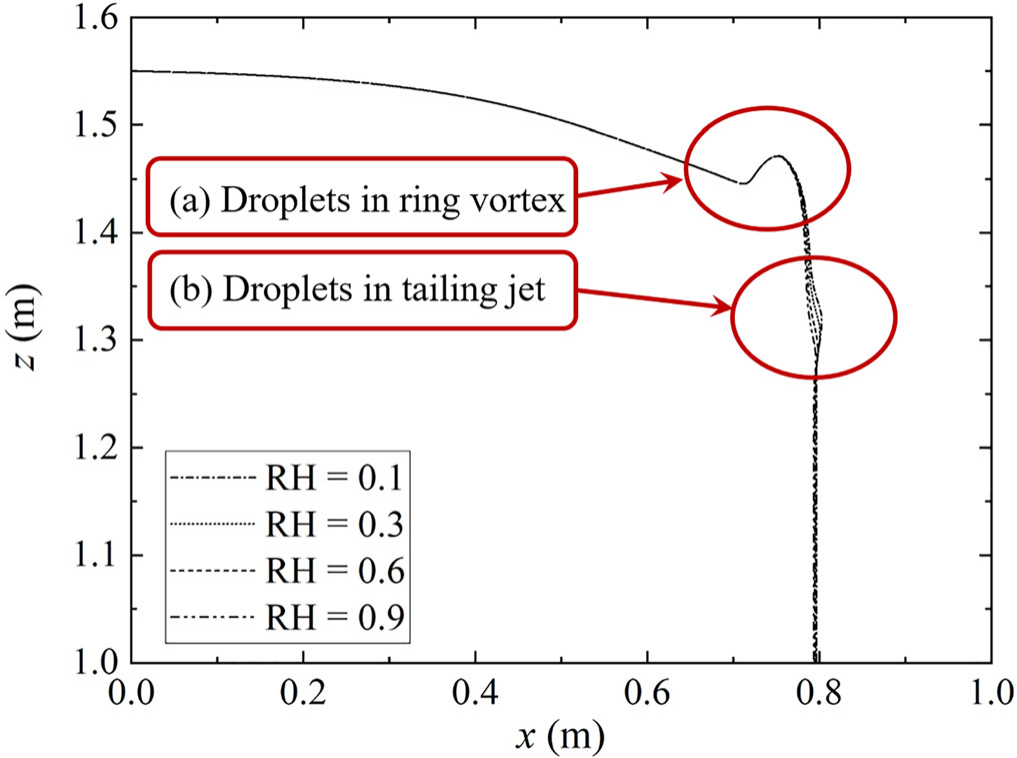
The influence of different humidity fields on the trajectory of droplets. The ambient air temperature and the initial droplet temperature is 36.0 °C and 30.0 °C, respectively. The expiratory velocity is 5.5 m/s with *D*_0_ = 80.0 *μ*m. (a) indicates droplets in the ring vortex, (b) indicates droplets in tailing jet.

The influence of the humidity field on the dynamic trajectory of droplets can be observed in Fig. 8, and the dynamic transport process of droplets under the humidity field is reflected in Fig. 6. The droplets roll into the ring vortex under the combinational effect of inertial force and gravity. The droplets are wrapped in the saturated humidity field of the steam mass for protection, and the environmental humidity cannot affect the evaporation of the droplets [Fig. 8(a)]. The position and velocity direction of the droplet move forward and rotate with the ring vortex, and they separate finally [Figs. 6(b) and 6(c)]. At this time, the diameter of the droplets changes due to evaporation, and the smaller droplets [for RH = 0.1 in Fig. 8(b)] are more likely to enter the tailing jet under a small smaller gravitational effect. The shrinking jet only pushes the droplet forward Fig. 6(d). Under the strong protection of the humidity field, the droplets are exposed to the air within a very short time, because of the difference in their droplet dynamics.

### C. Effect of ambient air temperature

**FIG. 9.**
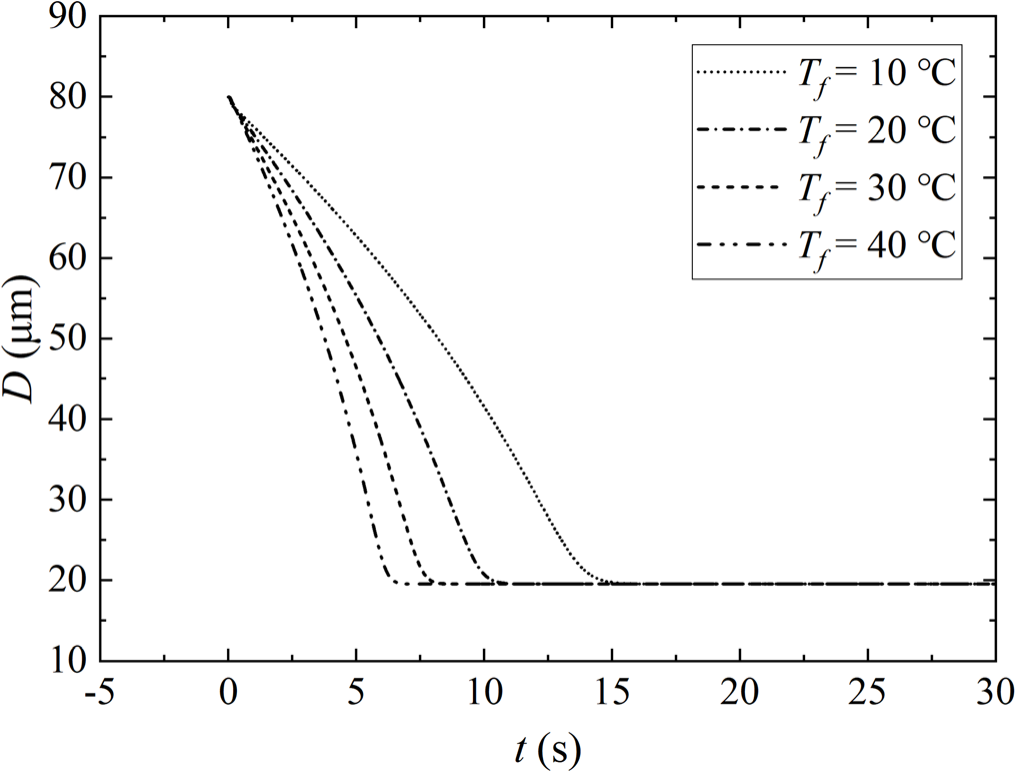
The evaporation rate for airborne sputum droplets with solid fraction at different air temperatures. The initial droplet temperature and the initial droplet size are 30.0 °C and 80.0 *μ*m, respectively. The expiratory velocity is 5.5 m/s with RH = 0.5.

Ambient air temperature is another important factor, which affects the evaporation and flow of aerosols. Ambient air temperature determines the saturation of water vapor pressure on the surface of the droplet, and the amount of heat exchange between the dispersed and continuous phase.^37^ Here, four different values of ambient air temperature are compared, namely, 10.0 °C, 20.0 °C, 30.0 °C, and 40.0 °C. The diameter of the initial droplet, the RH, and the velocity of exhaled airflow are 80.0 *μ*m, 0.5, and 5.5 m/s, respectively. The ambient air temperature accelerates the evaporation of droplets which is observed in Fig. 9. Droplets suspend evaporation at *t *=* *7.5 s for *T_f_* = 40.0 °C, or *t *=* *16.5 s for *T_f_* = 10.0 °C. The combination of Figs. 5 and 9 shows that the fraction of salt and RH determines the final diameter of the droplet.

**FIG. 10.**
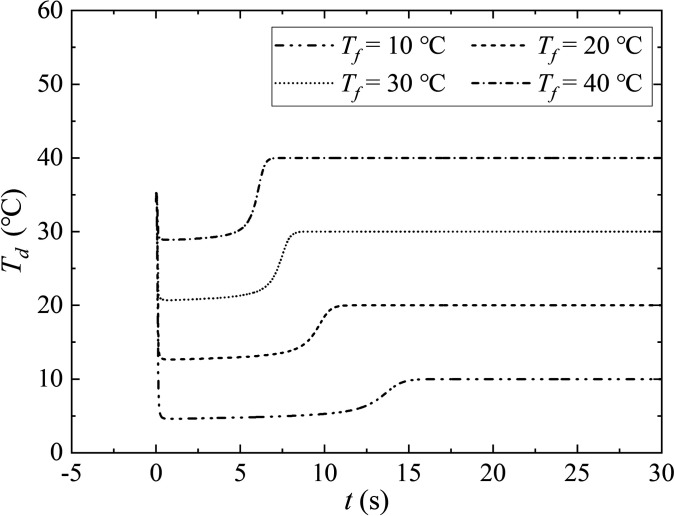
The evolution of droplet temperature with different ambient air temperature during evaporation. The initial droplet temperature and the initial droplet size are 36.0 °C and 80.0 *μ*m, respectively. The expiratory velocity is 5.5 m/s with RH = 0.5.

The evolution of droplet temperature with different air temperatures during evaporation is shown in Fig. 10. In the initial stage (*t *<* *0.16 s), the droplets are wrapped in turbulent clouds, and the droplets are almost unaffected. As the exhalation is completed, the droplet gradually evaporates. Both the convective heat exchange based on the difference between droplet temperature and air temperature, and the evaporative heat exchange caused by droplet evaporation, takes away the internal energy. Based on that, the temperature gradually drops. Then, the amount of the droplet evaporates into the air, and tends to be stable, as the result, the temperature is equilibrated. Finally, due to the presence of solid components, the droplet evaporates slowly and transforms into aerosols which suspended in the air. The temperature of the aerosols gradually becomes the same as that of the ambient air.

### D. Dynamic behavior of droplets with different sizes in humidity field

#### 1. Basic behaviors in humidity field

Saliva droplets are ejected from the mouth in different sizes.^38^ The distribution of droplet size predominately determines the time of evolution, the distance that they are free to move, and ultimately the risk for virus infection. The Rosin–Rammler distribution,^32^ also known as the Weibull distribution, is widely used to describe the size distribution of droplets ejected from the mouth.^12,13,16^ It is expressed as follows:

$$f(r)=\frac{qr^{q-1}}{{\bar{r}}^{q}}\text{exp}\;[-{\left(\frac{r}{\bar{r}}\right)}^{q}],$$ (25)

where *q* is an exponential factor that represents the width of the particle diameter distribution. $\bar{r}$ is the characteristic particle size, and given here as the average particle diameter.

**FIG. 11.**
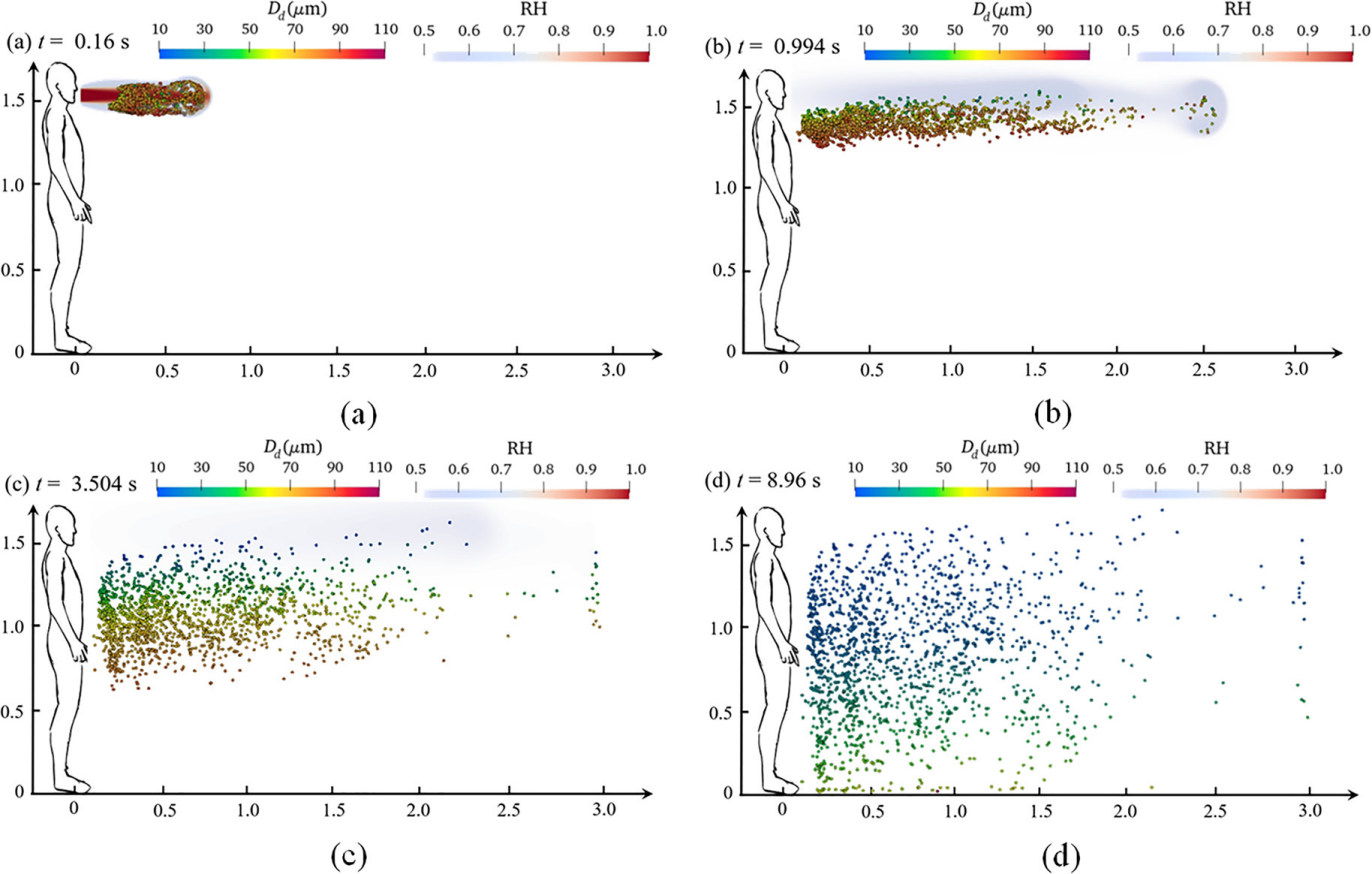
Dynamic dispersion of saliva droplet cloud with humidity field. The total number of ejected saliva is 1500 and obeys the Rosin–Rammler distribution with $\bar{r}$= 80.0 *μ*m. Each droplet is composed of 99.2379% water and 0.7621% salt. The outdoor ambient temperature, pressure, and relative humidity are 30.0 °C, 1.0 atm, and 0.5, respectively. The mouth temperature and the initial droplet temperature are both 36.0 °C. (a) *t *=* *0.16; (b) *t *=* *0.994; (c) *t *=* *3.504; and (d) *t *=* *8.96s.

**FIG. 12.**
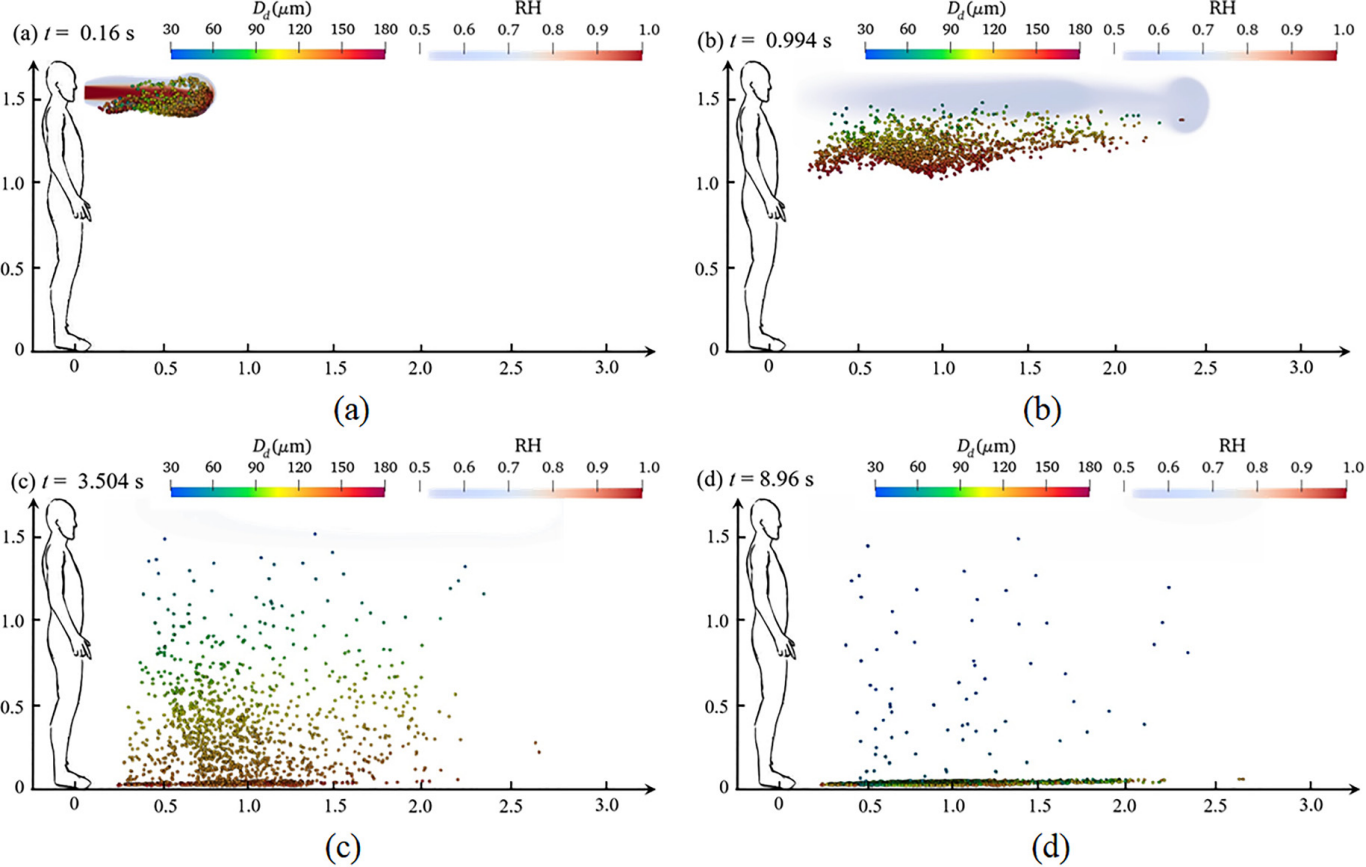
Dynamic dispersion of saliva droplet cloud with humidity field. The total number of ejected saliva is 1500 and obeys the Rosin–Rammler distribution with $\bar{r}$ = 140.0 *μ*m. Each droplet is composed of 99.2379% water and 0.7621% salt. The outdoor ambient temperature, pressure, and relative humidity are 30.0 °C, 1.0 atm, and 0.5, respectively. The mouth temperature and the initial droplet temperature are both 36.0 °C. (a) *t *=* *0.16; (b) *t *=* *0.994; (c) *t *=* *3.504; and (d) *t *=* *8.96 s.

**FIG. 13.**
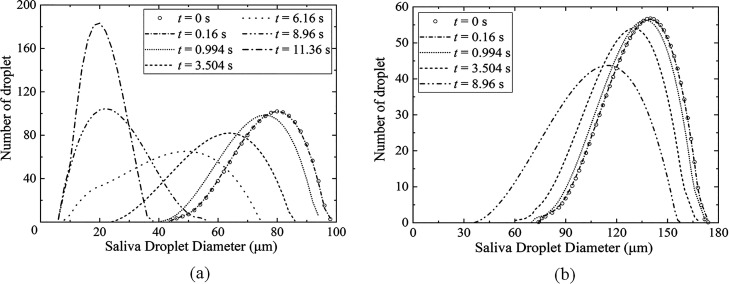
The droplet cluster size distribution at different moments. The initial droplets obey the Rosin–Rammler distribution. (a) average diameter 80.0 *μ*m; and (b) average diameter 140.0 *μ*m.

Coughing, sneezing, and exhalation all involve the accumulation of air pressure in the lungs, which forms a powerful jet of air, and its duration can reach from 0.01 s to 0.25 ms.^12^ This evolution refers to the transportation, evaporation, and heat transfer of saliva droplets during coughing. Figures 11 and 12 show the dynamic dispersion of saliva droplets with different average diameters (80.0 and 120.0 *μ*m, respectively) on the humidity field during the entire sneezing cycle from mouth to floor. Figure 13 shows the droplet cluster size distribution at different moments with different average diameters (80.0 and 120.0 *μ*m, respectively). The total number of ejected saliva is 1500 and obeys the Rosin–Rammler distribution. Each droplet is composed of 99.2379% water and 0.7621% salt. The outdoor ambient temperature, pressure, and relative humidity are 30.0 °C, 1.0 atm, and 0.5, respectively. The mouth temperature and the initial droplet temperature are both 36.0 °C. In the initial process of exhalation (0–0.16 s), the droplets ejected from the lips move almost linearly along the initial velocity under the effect of the inertia force and the drag force [Figs. 11(a) and 12(a)], and the droplet moves to a distance of 0.7 m. Most droplets are wrapped into the exhaled turbulent airflow. The humidity of the turbulent cloud is close to saturation, and the temperature crystallizes at human body temperature (36.0 °C). The droplet only has the motion of dispersion, without evaporation and heat transfer. Therefore, Fig. 13 shows that the droplet size distribution is the same as that of initial state.

After the jet is exhaled, airflow velocity gradually decreases, and inertial force is gradually replaced by drag force and gravity. The larger droplet is dominated by gravity and sink gradually. Their transport distance in the x-direction maintains as 1.0 m, while the smaller droplet disperses as far as 2.5 m away under the effect of the drag force and the Brownian force [Figs. 11(b) and 12(b)]. The most obvious evidence appears in Figs. 11(c) and 11(d) and 12(c) and 12(d). Under the same conditions, droplets with an average diameter of 140.0 *μ*m settled on the ground, while an average diameter of 80.0 *μ*m was suspended in the air. At the same time, the humidity and temperature fields gradually merge with the air, and their values continuously decrease until they are consistent with those of the environment [Figs. 11(b) and 12(b)]. The difference between the surface temperature of the droplet and the ambient temperature gradually increases, and the humidity evolution has a similar situation. Then, the droplet begins to evaporate and transfer heat. Figures 13(a) and 13(b) show that the overall droplet distribution value moves to the left, and its peak value decreases. Such as in Fig. 13(a), the largest droplet diameter decreases from 100.0 *μ*m at 0.16 s to 75.0 *μ*m at 6.16 s, and the average droplet diameter distribution also gradually reduces from 80.0 to 50.0 *μ*m.

Figures 11(c) and 11(d) show that the phenomenon of droplet stratification occurs in the equilibrium stage of the deposition process of saliva droplets. Under the effect of gravity, the larger droplets fast sink, while the smaller droplets suspend and wander through the air because of the Brownian motion. Meanwhile, the exhaled airflow is fused with the air, and the humidity field and temperature field could not hinder the droplet evaporation anymore. From 6.16 to 11.36 s, as seen in Fig. 11, the minimum droplet size on the left side of the curve no longer changes, and the peak of the droplet size distribution grows, which shows a different phenomenon from the previous drop.

As the water evaporates, the proportion of nonvolatile solid components in the droplets continues to increase, and the water activity decreases. The droplets tend to be saturated, and formed as aerosols, which are suspended in the air. Most of the droplets in Fig. 12 settles on the ground before evaporation is completed.

The maximum horizontal distance, traveled by the droplet transmission route and the pattern of deposition, are important factors to evaluate infection risk. The dynamic dispersion of the saliva droplet cloud with the humidity field and different sizes can be observed in Figs. 11(d) and 12(d), which accurately presents the maximum deposition area and settling velocity. For various droplet sizes, the deposition mode changes significantly. For an average droplet diameter of 140.0 *μ*m, more than 50.0% of the droplets settle on the ground at 3.504 s, and the horizontal maximum value of the traveled distance is only 2.5 m. By contrast, for an average droplet diameter of 80.0 *μ*m, all droplets suspend in the air at the same time (3.504 s), and the horizontal maximum value of the traveled distance reaches 3.0 m.

The sedimentation patterns in these size ranges clearly show that the effect of gravity and i the Brownian force is dominant, and the influence of the flow field is greatly reduced. When reducing the size distribution, this pattern becomes chaotic. For example, droplets with an average diameter of 80 *μ*m only sank 0.8 m, and most droplets are still suspended in the air. Under the effect of the Brownian force and the Stokes drag force, some of the droplets move a longer distance, reaching 3.0 m. The present results indicate that with the decrease in droplet size distribution, the effect of inertial force and gravity determining its trajectory is decreasing. Relatively speaking, the influence of environmental conditions and aerodynamic resistance increases for small droplets. The area of contamination, particularly the maximum contaminated longitudinal distance, becomes larger.

#### 2. Dynamic behavior of droplets during humidity field evolutions

A key feature of the present simulation is the coupling of transport phenomena, evaporation flow, and droplet dynamics. To test the influence of the evaporated droplets on sedimentation distance in humidity, a comparative study was conducted on the ejection of seven droplets with initial diameters of 20.0–140.0 *μ*m. The ambient air temperature and the initial droplet temperature is 36.0 °C and 30.0 °C, respectively. The dynamic trajectory of droplet transport near the humidity field is shown in Fig. 15. The lightest droplets (*D_d_* = 20.0 *μ*m) rebound to a higher position than the initial position [Fig. 16(f)].

**FIG. 14.**
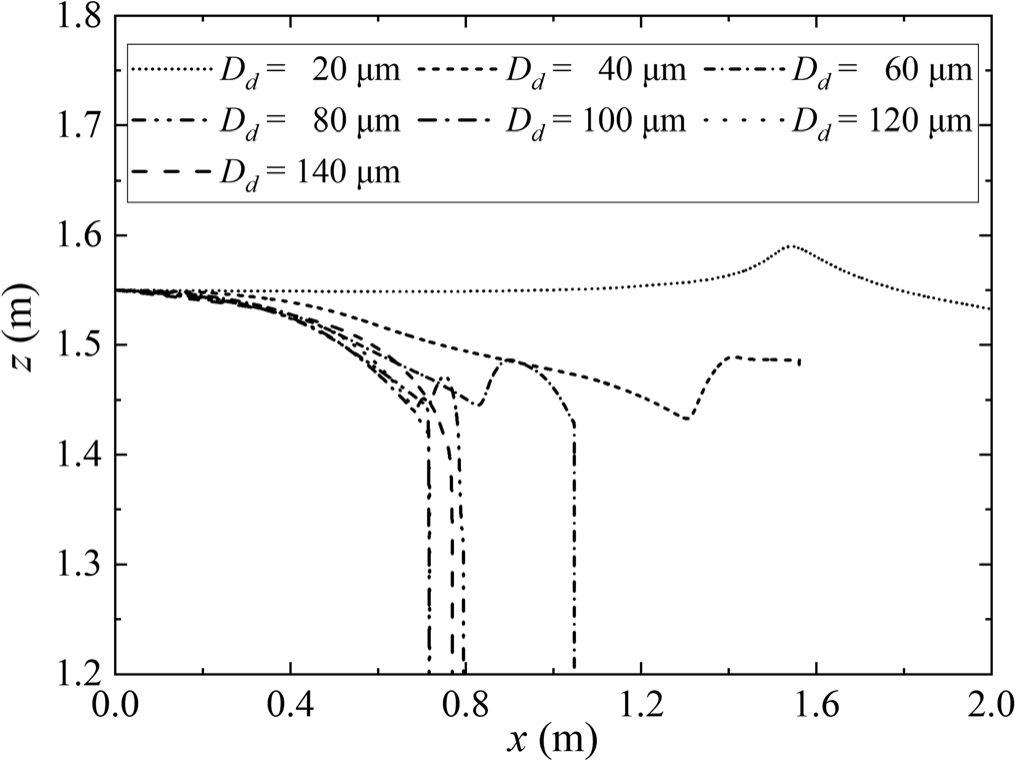
The dynamic trajectory of droplet transport near the humidity field. The ambient air temperature and the initial droplet temperature are 36.0 °C and 30.0 °C, respectively. The expiratory velocity is 5.5 m/s. The initial diameters of the droplets are 20.0–140.0 *μ*m, respectively.

Although gravity does not significantly affect the dynamic trajectory of a droplet, it does cause a slight asymmetry between the trajectories of particles released above and below the centerline. At the same time, relative to droplets that settle smoothly (with diameters of 120.0 or 140.0 *μ*m), medium (with diameters of 40.0, 60.0, 80.0, or 100.0 *μ*m) droplets rebound to the original height after settling for a period (Fig. 14). This regular rebound seems to completely resist the gravity of the droplet itself.

**FIG. 15.**
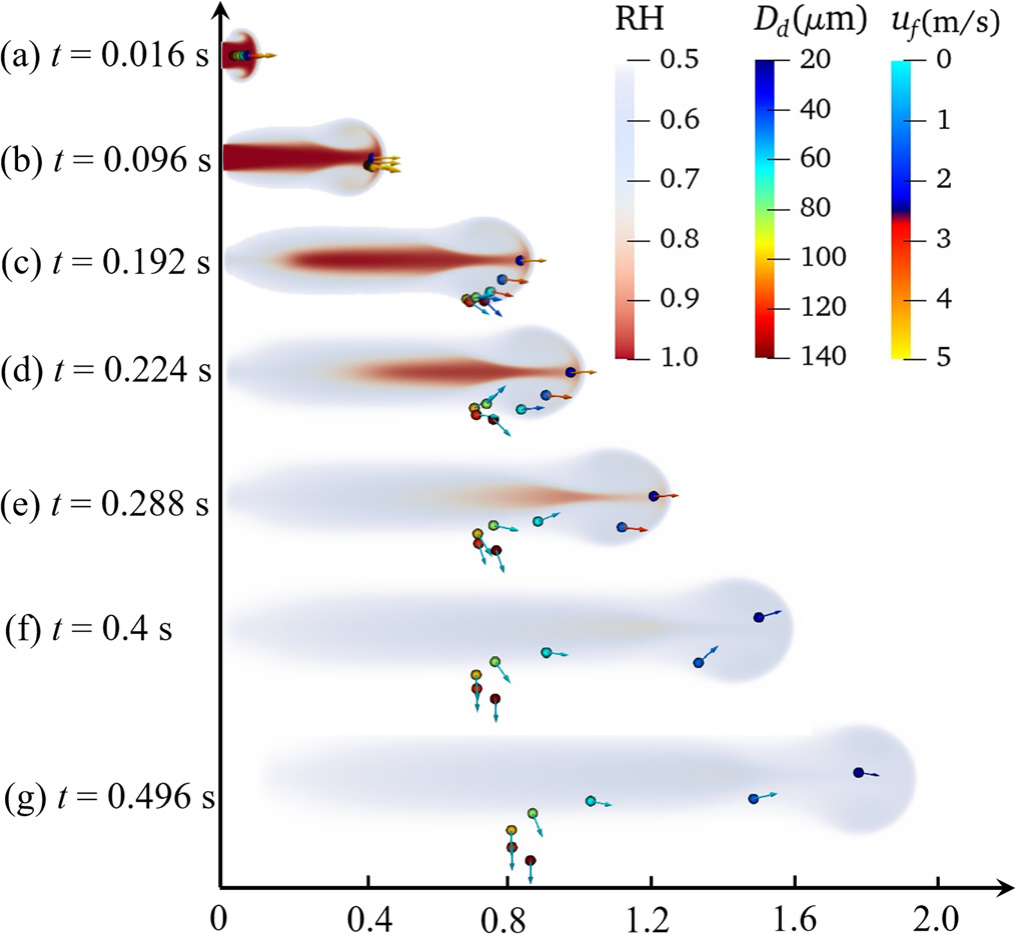
The time history of evaporating droplets near the vortex with humidity field. The ambient air temperature and the initial droplet temperature are 36.0 °C and 30.0 °C, respectively. The expiratory velocity is 5.5 m/s. The initial diameters of the droplets are 20.0–140.0 *μ*m, respectively. (a) *t *=* *0.016; (b) *t *=* *0.096; (c) *t *=* *0.192; (d) *t *=* *0.224; (e) *t *=* *0.288; (f) *t *=* *0.4; and (g) *t *=* *0.496 s.

To explain the rebounding phenomenon, the dynamic trajectory of droplet transport near the humidity field is presented in Fig. 15. When the large droplet (120.0 or 140.0 *μ*m) wrapped in a humidity field, the evaporation does not significantly change the mass before it settles. After the inertial force is exerted to its extreme [Fig. 15(c)], the droplet is less responsive to the flow field due to gravitation, and as a result, they settle quickly and disperse at shorter distances (0.8 m). Therefore, the large droplets are not affected by the cloud dynamics and show astringent settlement. However, the smaller droplet (20.0 *μ*m) is affected by the upward vortex and are ejected further upstream as a result of their low weight and the disturbance of Brownian force and of buoyancy. A complete ring vortex rolled the droplets in the jet into the fluid, then rolls them out and moves forward. Their low gravity keeps them wrapped in the humidity field for a long time and they float further [reaching 2.0 m, see Figs. 15(g), 11(d), and 12(d)]. The medium droplet (20.0–100.0 *μ*m) appears from following the similar process, but the difference is that the weight is double for droplets with a diameter of 20.0 *μ*m, so the influence of gravity is greater than that of the Brownian motion, and the droplet is drawn into the lower vortex. The medium droplets rebound to the original height after settling for a period [Figs. 16(c)–16(g)], which can be considered the buffer time for evaporation (shrinking the droplet diameter). Therefore, this is the root cause of their airborne suspended [Fig. 11(d)]. These results indicate that 2.0 m social distance may not safe and should be increased to around 4.0 m. It is necessary to wear a mask in public indoor spaces in areas with widespread transmission of SARS-CoV-2.

## IV. CONCLUSIONS

An evaporation flow model of airborne sputum droplets that considers the humidity field evolution and solid fraction interactions has been proposed in this study. This model features an advection-diffusion equation that describes the evolution of a supersaturation field. The numerical model has been applied to reveal the mechanism of saliva particles transport in a humidity field.

(1) The newly proposed physical and numerical model show good accuracy compared with existing models of airborne sputum droplets systems.
(2) It is found that the humidity field generated from the respiratory tract plays a very important role in the evaporation and transport of droplets. With droplets (80.0 *μ*m) evaporated at different humidity values (0.1 and 0.9), the final (equilibrium) diameter differed (16.3 and 29.8 *μ*m, respectively) and the time consumption is 8 times difference (4.0 and 32.0 s, respectively). However, the difference in droplet diameter can reach 5.0 *μ*m with RH = 0.1 and the size distribution is nearly the same within 0.16 s of exhalation.
(3) Increasing airborne temperatures can accelerate the evaporation and the salt content and relative humidity parameter jointly define the equilibrium diameter of the aerosol. For droplets of the same size (80.0 *μ*m) that evaporate at different temperatures (10.0 °C and 40.0 °C), the time consumptions of 7.5 and 16.5 s, respectively.
(4) Droplets of the same diameter have different dynamics at the front end of the vortex and the back end of the jet due to the different effects of different values of airborne humidity. Under the accelerated evaporation of the humidity (with a shrinking droplet diameter), light droplets are propelled by the tailing jet. Vortex dynamics of droplets in a humidity field is sensitive to the size parameter, while small and medium-sized droplets (20.0–80.0 *μ*m) fall into the rotation of the vortex and rebound backward, which is the buffer time for evaporation (shrinking droplet diameter). This leads to the suspension of more droplets in the environment. Small droplets (20.0 *μ*m) are suspended in a higher position under the rotation of the vortex, which penetrates the respiratory tract more easily.

## SUPPLEMENTARY MATERIAL

See the supplementary material for detailed data that supports the findings of humidity field evolution and droplets distributions in this study.

## APPENDIX: CALCULATION OF THE PHASE SATURATION PARAMETERS

The physical variables appearing in this model will be listed in this Appendix. The saturated vapor pressure (*P_sat,T_* [Pa]) determined by temperature can be written as

$$P_{\mathrm{sat},T}=6.1078\times 10^{\frac{7.5T}{\left(T+237.3\right)}},$$
(A1)

where *T* (°C) could represent ambient air temperature *T_f_* (°C) and droplet temperature *T_d_* (°C), respectively.

The diffusion coefficients of water vapor *D* in airborne at normal pressure for various water/air temperatures can be calculated by^39^

$$D=22.5\times 10^{-6}{\left(\frac{T}{273.15}\right)}^{1.8}.$$
(A2)

The density and viscosity coefficient of air can be calculated by a function of temperature as

$$\rho =\frac{351.99}{T}+\frac{344.84}{T^{2}},$$
(A3)

$$\mu =\frac{1.4592\times 10^{-6}T^{3/2}}{109.1+T}.$$
(A4)
